# Stochastic Petri net model describing the relationship between reported maternal and congenital syphilis cases in Brazil

**DOI:** 10.1186/s12911-022-01773-1

**Published:** 2022-02-15

**Authors:** Ricardo A. M. Valentim, Gleyson J. P. Caldeira-Silva, Rodrigo D. da Silva, Gabriela A. Albuquerque, Ion G. M. de Andrade, Ana Isabela L. Sales-Moioli, Talita K. de B. Pinto, Angélica E. Miranda, Leonardo J. Galvão-Lima, Agnaldo S. Cruz, Daniele M. S. Barros, Anna Giselle C. D. R. Rodrigues

**Affiliations:** 1 Laboratory of Technological Innovation in Health, Federal University of Rio Grande do Norte, Natal, Brazil; 2 Public Health School of Rio Grande do Norte, Natal, Brazil; 3 Postgraduate Program in Infectious Diseases, Federal University of Espírito Santo, Vitória, Brazil; 4 Digital Metrópole Institute, Federal University of Rio Grande do Norte, Natal, Brazil

**Keywords:** Stochastic Petri net, Congenital syphilis, Maternal syphilis

## Abstract

**Introduction:**

Syphilis is a sexually transmitted disease (STD) caused by Treponema pallidum subspecies pallidum. In 2016, it was declared an epidemic in Brazil due to its high morbidity and mortality rates, mainly in cases of maternal syphilis (MS) and congenital syphilis (CS) with unfavorable outcomes. This paper aimed to mathematically describe the relationship between MS and CS cases reported in Brazil over the interval from 2010 to 2020, considering the likelihood of diagnosis and effective and timely maternal treatment during prenatal care, thus supporting the decision-making and coordination of syphilis response efforts.

**Methods:**

The model used in this paper was based on stochastic Petri net (SPN) theory. Three different regressions, including linear, polynomial, and logistic regression, were used to obtain the weights of an SPN model. To validate the model, we ran 100 independent simulations for each probability of an untreated MS case leading to CS case (PUMLC) and performed a statistical *t*-test to reinforce the results reported herein.

**Results:**

According to our analysis, the model for predicting congenital syphilis cases consistently achieved an average accuracy of 93% or more for all tested probabilities of an untreated MS case leading to CS case.

**Conclusions:**

The SPN approach proved to be suitable for explaining the Notifiable Diseases Information System (SINAN) dataset using the range of 75–95% for the probability of an untreated MS case leading to a CS case (PUMLC). In addition, the model’s predictive power can help plan actions to fight against the disease.

## Background

Syphilis is a sexually transmitted disease whose etiologic agent, first identified in 1905, is the bacterium *Treponema pallidum* [1]. According to the World Health Organization (WHO), over 6 million new syphilis cases are reported each year [2]. The disease is mainly transmitted through unprotected sexual intercourse (acquired syphilis), although it can also be transmitted from mother to child during pregnancy (congenital syphilis) [3–6]. Exposure to syphilis in the intrauterine environment can culminate in severe implications with adverse pregnancy outcomes in more than 50% of cases, such as miscarriage, stillbirth, neonatal death, and early and late complications in live births [7, 8].

Congenital syphilis (CS) cases are strongly associated with inadequate prenatal care (PC). This mainly results from inadequate screening and inadequate or lack of treatment for the disease in pregnant women and their partners. Delayed detection of the condition in pregnant women plays a critical role in disease progression—the earlier a pregnant woman receives treatment in gestation, the greater the likelihood of the fetus not becoming infected. Untreated or inadequately treated pregnant women can transmit *T. pallidum* to their babies at any stage of pregnancy through the placenta or during childbirth [3–6]. Although simple and simultaneous treatment is available for the mother and fetus in PC; in 2016 alone, there were 661,000 estimated new cases of CS. CS is currently the second leading cause of preventable stillbirths worldwide [9, 10].

In Brazil, syphilis has been declared an epidemic due to its high morbidity and mortality rates, principally affecting pregnant women and newborns [11]. In a time series analysis, from 2013 to 2018, the number of acquired syphilis cases in Brazil surged by nearly 404.34%. In addition, maternal syphilis (MS) and congenital syphilis (CS) cases increased by 302.23% and 189.24%, respectively, in the same span [12, 13]. Such data suggest a worrisome vulnerability regarding maternal and child health in Brazil [14].

Preliminary epidemiological analyses have indicated that MS is more prevalent in women living under precarious socioeconomic conditions, consequently unveiling greater social and gender inequalities and women’s reproductive vulnerability, making it challenging to control syphilis in this population [15]. In this context, developing strategies to identify vulnerable populations and thus offer diagnostic tests and adequate treatment for pregnant women who test positive during PC and their sexual partners would likely break the cycle of CS transmission.

In an analysis of global data, the ratio of congenital to MS case notifications (RCMCN) was 75% in 2012 and 66% in 2016, while the RCMCN for the respective years in Brazil was 72% and 59%; that is, the reduction in the proportion of CS cases to MS cases worldwide and in Brazil stands out [16, 17]. However, when investigating the relationship between reported cases of MS and CS, it is important to emphasize that the reported cases do not necessarily represent the actual incidence of the disease in the population. Factors such as access to diagnosis and the integrity of the reported data are extremely relevant when representing the epidemiological context of the disease. However, notification-based analyses make it possible to adopt effective preventive interventions [18].

That being said, the present study aimed to mathematically describe the relationship between maternal and congenital syphilis case notifications in Brazil, considering the likelihood of diagnosis and adequate maternal treatment during the prenatal period using stochastic Petri nets (SPNs). This method allows for mathematical representation, in addition to enabling the verification of the properties and correctness of the system described from its analysis mechanisms [15].

## Methods

The development of the present study consisted of the following five steps: (1) Data characterization, which described the data source and the corresponding period of the dataset; (2) Building an SPN framework, which explained the model construction process; (3) Analytical calculations of the probabilities associated with the transitions, which described the calculation process of the SPN transitions; (4) Regressions, which detailed the regression methods that were used for data analysis; and finally, (5) Simulation evaluation, which described the SPN simulation process. The following subsections detail what was accomplished in each step.

### Data characterization

The data used for analysis were obtained from the Notifiable Diseases Information System (SINAN) [11], developed by the Brazilian Ministry of Health (MoH) [11, 19, 20]. Our database covers the span from 2010 to 2020 and comprises two datasets: the first refers to cases of syphilis in pregnant women, and the second refers to CS cases.

Of note, it was not possible to connect individual records in both datasets since all the information that could allow for this was removed in the anonymization process. However, we established a common temporal reference for both datasets to relate these two occurrences. For the CS case dataset, we used an annual aggregation considering a child’s date of birth rather than the date of notification. For MS cases, we used the recorded gestational period to estimate the date of the pregnancy outcome based on a 40-week gestation. Once a common date was established, we could compare the records of both datasets in the time domain. In addition, in the case of the CS dataset, we also used two attributes: the date of maternal diagnosis and the status of the treatment provided to the mother.

Although this information was found in the CS dataset, it referred to the information about the mothers. Regarding the time of diagnosis, the possible values for the field were diagnosis during PC, during childbirth, after childbirth, not included, or not reported. The other field regarded treatment, which assumed different values: adequate treatment, inadequate treatment, no treatment, and not reported. With these two attributes in the CS dataset, it was possible to establish three different groups:

- CS cases whose mothers were not diagnosed during PC and had no opportunity for treatment;
- CS cases whose mothers were diagnosed during PC and received adequate treatment;
- CS cases whose mothers were also diagnosed during PC but did not receive adequate treatment.

Accordingly, Table 1 summarizes the SINAN data used in this paper, considering the cases of MS and the different groups of CS cases. Using the same assumptions, we were able to infer subsets of MS data to establish a cause-and-effect relationship. Then, from the number of MS cases in one year, we allotted two disjoint sets based on the date a pregnant woman was diagnosed, thus creating a new layer in our analysis. Finally, starting from the subset of MS cases where the diagnosis was made during PC, we created another layer based on the treatment received. From that, we established four subsets:

- The subset of MS cases diagnosed during PC;
- The complementary subset of MS cases without a diagnosis during PC;
- The subset of MS cases where the diagnosis was made during PC and the treatment received was adequate;
- The subset of MS cases where the diagnosis was made during PC and the treatment received was inadequate.

**Table 1:** Summary of the SINAN data used in this study, considering the reported MS cases and the different groups of reported CS cases

Calendar year	MS reports $\left( {p_{0} } \right)$	CS reports with adequate maternal treatment during PC $\left( {p_{7} } \right)$	CS reports without adequate maternal treatment during PC $\left( {p_{9} } \right)$	CS reports without a maternal diagnosis during PC $\left( {p_{5} } \right)$	CS case reports $\left( {p_{7} + p_{9} + p_{5} } \right)$
2010	9542	203	2688	4088	6979
2011	12,941	226	3825	5517	9568
2012	16,041	266	4689	6749	11,704
2013	19,479	304	5995	7725	14,024
2014	25,029	443	7499	8446	16,388
2015	30,851	610	9543	9566	19,719
2016	36,023	703	11,528	9066	21,297
2017	46,192	983	13,441	10,605	25,029
2018	60,830	1287	13,945	11,220	26,452
2019	62,562	1303	12,877	9965	24,145
2020	47,488	3312	5926	5766	15,004

**Fig. 1:**
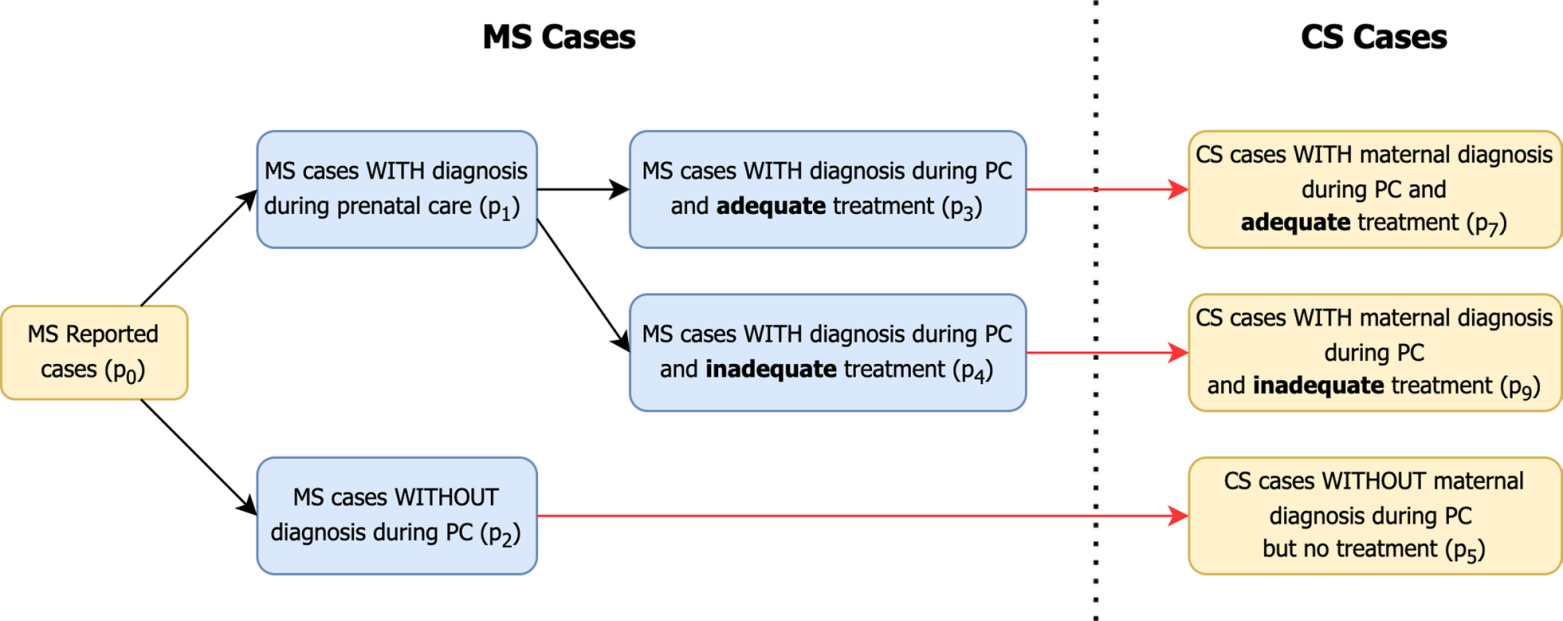
Flowchart of the relationship between the reported cases of MS and CS considering maternal diagnosis and treatment

This logical structure used in the mother-to-child transmission flowchart is depicted in Fig. 1, and it was also used in the SPN modeling. The indications inside the parentheses are references to places in the stochastic Petri net framework, discussed in the following subsection.

### Building an SPN framework

The occurrence of CS cases seems to depend on MS and several other factors, making it very difficult to accurately predict. Hence, considering the (1) screening and (2) treatment of pregnant women during PC, it is possible to build a model describing the relationship between reported cases of maternal and congenital syphilis through graphic notation in stochastic Petri nets. These are suitable for presenting a formal description of dynamic behavior in a complex system.

Mathematically, a stochastic Petri net (SPN) [21–23] is defined as six-tuple $SPN = \left\{ {P,T,F,M,\Lambda ,W} \right\}$, where:

- $P = \left\{ {p_{1} ,p_{2} ,p_{3} , ..., p_{m} } \right\}$ is a finite set of places;
- $T = \left\{ {t_{1} , t_{2} , t_{3,} , ..., t_{n} } \right\}$ is a finite set of transitions;
- $F \subseteq \left( {P \times T} \right) \cup \left( {T \times P} \right)$ is a set of arrows;
- $M:P \to N$ where $m_{1} = M\left( {p_{i} } \right)$ is a marking whose ith component represents the number of tokens in the ith place;
- $\Lambda :T \to \left[ {0,1} \right]$ where $\lambda_{i} = \Lambda \left( {t_{i} } \right), i = 1,2,3,...,n$ is a set of firing rates associated with the transitions,and finally,
- $W:F \to N$* is a set of weights associated with the arrows.

Graphically, an SPN place is represented by a circle, a transition by a bar, and an arrow that joins a place to a transition or vice versa. Between a place $p_{m}$ and $p_{n}$, there is always an arrow-transition-arrow sequence. No arrow connects two places; neither does an arrow connect two transitions. A place is said to be an input place if it precedes any transition where an arrow originates, and likewise, it is considered an output place when it transitions where an arrow is intended.

Finally, a token represents the occurrence of an action that will be consumed by a transition and will remain in one place for a given range of interactions. A place can hold one or more tokens, and a transition will only be triggered when its input place contains the minimum number of tokens required [24]. The transition firing is atomic, i.e., tokens are removed from input places and put into output places with a single, indivisible operation. One of the advantages of Petri nets is their intuitive graphical representation, which is particularly helpful in the phases of the development of a biological system model and the simulation of the system’s behaviour [25, 26].

There are two complementary subparts in an SPN, namely, the static and the dynamic elements. First, the static sets relate to a net’s design: places, transitions, and arrows. Second, the dynamic elements include the tokens associated with each place, the firing rates associated with each transition, and finally, the weights of each arrow.

**Fig. 2:**
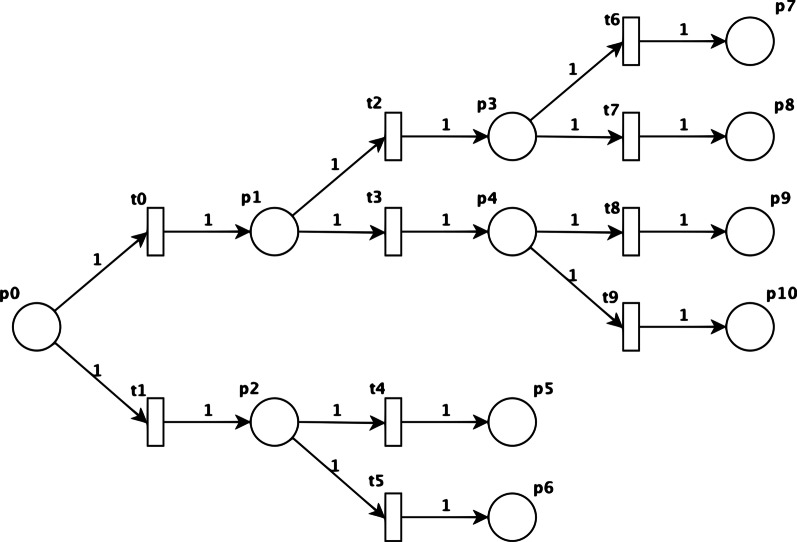
Graphical notation of the SPN considering the flowchart between MS and CS reported notifications

From what was discussed regarding Fig. 1, we had everything needed to design the framework of the SPN, as shown in Fig. 2. It is noteworthy that this graphical representation adds three more branches when compared to Fig. 1. This is because the number of tokens was constant, and therefore, the MS cases that did not lead to CS cases should be encompassed.

Once the structure was defined, it was necessary to establish the initial conditions for the SPN model. Thus, we needed to define $M$, that is, the set of numbers of tokens associated with $P$, where $m_{0}$ starts as the number of MS cases in one year, and for the other elements from $m_{1}$ to $m_{10}$, the initial value was 0. For set $W$, all weights were equal to 1. Set of places $P$ where $M:P \to N, m_{i} = M\left( {p_{i} } \right)$ are mapped in Table 2.

**Table 2:** Description of the places mapped on the stochastic Petri net (SPN)

Place $\left( {p_{i} } \right)$	Tokens $\left( {m_{i} } \right)$	Tokens $m_{i}$ in the place $p_{i}$ represents
$p_{0}$	$m_{0}$	Number of reported cases of MS in one year
$p_{1}$	$m_{1}$	Number of MS cases diagnosed during prenatal care
$p_{2}$	$m_{2}$	Number of MS cases undiagnosed during PC
$p_{3}$	$m_{3}$	Number of MS cases with screening and adequate treatment during PC
$p_{4}$	$m_{4}$	Number of MS cases diagnosed during PC but without treatment
$p_{5}$	$m_{5}$	Number of CS cases without maternal diagnosis during PC and without treatment
$p_{7}$	$m_{7}$	Number of CS cases with maternal diagnosis and adequate treatment during PC
$p_{9}$	$m_{9}$	Number of CS cases with maternal diagnosis and inadequate maternal treatment during PC
$p_{6} ,p_{8} ,p_{10}$	$m_{6} ,m_{8} ,m_{10}$	Number of MS cases that did not lead to CS cases

Finally, the set of firing rates associated with the transitions $\left( \Lambda \right)$ was calculated as shown in the following subsection. Therefore, the model had two inputs, the number of MS cases and the firing rate set $\Lambda$, and one output, the number of CS cases in the same period. The set of transitions $\Lambda$ is mapped in Table 3.

**Table 3:** Descriptions of the transitions mapped on the stochastic Petri net (SPN)

Transition $\left( {t_{j} } \right)$	Firing rate $\left( {\lambda_{j} } \right)$	Firing rate $\lambda_{j}$ associated with $t_{j}$ represents
$t_{0}$	$\lambda_{0}$	Probability of a case report of MS (p_0_) being diagnosed during prenatal care
$t_{1}$	$\lambda_{1}$	Probability of a case report of MS (p_0_) being diagnosed during PC
$t_{2}$	$\lambda_{2}$	Probability of a pregnant woman diagnosed during PC receiving adequate treatment
$t_{3}$	$\lambda_{3}$	Probability of a pregnant woman diagnosed during PC not receiving adequate treatment
$t_{4} , t_{8}$	$\lambda_{4} , \lambda_{8}$	Probability of an untreated case of MS leading to CS
$t_{5} , t_{9}$	$\lambda_{5} , \lambda_{9}$	Probability of an untreated case of MS not leading to CS
$t_{6}$	$\lambda_{6}$	Probability of a diagnosed and treated case of MS leading to CS
$t_{7}$	$\lambda_{7}$	Probability of a diagnosed and treated case of MS not leading to CS

### Analytical calculation of the probabilities associated with transitions

In this subsection, we used the static framework of the SPN and the mathematical notations previously specified. Observing the network structure made it possible to recognize that the transitions were organized in pairs, thus representing the binary occurrence of an event. Therefore, the sum of the probabilities of each pair was always equal to 1. In this way, we could describe only one probability for each pair, as they are complementary. Thus, firing rates with odd indices obey the equation:

$$\lambda_{i} = 1 - \lambda_{i - 1} ,\quad {\text{where}}\;i = \{ 1, 3, 5, 7, 9\}$$

Another significant aspect was that the $\lambda_{4}$ and $\lambda_{8}$ firing rates represented the likelihood of a mother transmitting the infection to her baby in situations where she had not received adequate treatment during prenatal care (PUMLC), so we have $\lambda_{4} = \lambda_{8}$. Then, when we parameterized PUMLC, we could write $\lambda_{0} , \lambda_{2} ,$ and $\lambda_{6}$ as parametric functions of such a probability. The main focus of this subsection was on the calculations of $\lambda_{0} , \lambda_{2} ,$ and $\lambda_{6}$.

In position $p_{5}$, each token $m_{5}$ represented the number of CS cases whose mothers went undiagnosed during PC as a consequence of the number of mothers with syphilis who were not diagnosed in a timely manner. Thus, $m_{5} = m_{2} *\lambda_{4}$. Since we had the number of cases of CS where mothers who were not diagnosed during prenatal care $m_{5}$ coming from the dataset, and we assumed the value of $\lambda_{4}$—that is, the probability of an untreated MS case leading to a CS case (PUMLC)—we analytically inferred $m_{2}$.

From the cases of syphilis during pregnancy, represented by $m_{0}$ at position $p_{0}$, we could infer $m_{1}$ since $m_{0} = m_{1} + m_{2}$. Specifically, we divided the number of MS cases according to whether or the diagnosis was made during prenatal care.

Since the values of $m_{0}$ and $m_{1}$ were known, we could determine $\lambda_{0} = m_{1} /m_{0}$. Therefore, $\lambda_{0}$ represents the probability of a case report of a woman with MS being diagnosed during PC.

Through the same logic, we started from $m_{9}$; that is, the tokens in position $p_{9}$ represented the number of CS cases whose mothers were diagnosed with syphilis during PC but had not received adequate treatment. Therefore, we could write that $m_{9} = m_{4} *\lambda_{8}$, and in this way, $m_{4} = m_{9} /\lambda_{8}$. As a result, we then calculated $m_{3} = m_{1} - m_{4}$. Therefore, to calculate $\lambda_{2}$, the rate of mothers with syphilis who received adequate treatment, we determined $\lambda_{2} = m_{3} /m_{1}$.

The last rate we determined is $\lambda_{6}$, which represented the likelihood of an MS case leading to a CS case in situations where the mothers received a diagnosis and treatment during PC. Finally, we determined $\lambda_{6} = m_{7} /m_{3}$, which is from the dataset and represents the number of CS cases whose mothers received adequate treatment.

### Regressions for the simulation

Once the definitions for obtaining the initial conditions had been established, it is relevant to note that these calculations depended on the available data aggregated yearly and the range of probability of an untreated MS case leading to a CS case.

Consequently, we found a set of initial conditions for each year and each PUMLC. Nonetheless, as we sought to calibrate the SPN in order for it to serve as a tool for analyzing and predicting future case scenarios, we tried to find the initial configurations that required the fewest possible input parameters. Thus, in this subsection, we employed regression techniques to understand the behavior of the probabilities (firing rates) over time and therefore predict them in the future. For this, we considered the span from 2010 to 2018 for model training and 2019 and 2020 for testing.

For the probability of an untreated MS case leading to CS case (PUMLC) we tested a range of 60–100%. However, when we assumed values for PUMLC below 75%, we found a break in the cause-effect relationship between $m_{3}$ and $m_{7}$. Thus, the data could not be explained by the probability of an untreated MS case leading to a CS case below 75% and above 97% using the proposed model. Consequently, we used the values in the range of $\left[ {0.75, 0.95} \right]$ with variations of 5 percentage points. Therefore, in all experiments, we considered $PUMLC = \left\{ {0.75, 0.8, 0.85, 0.9, 0.95} \right\}$*.*

**Fig. 3:**
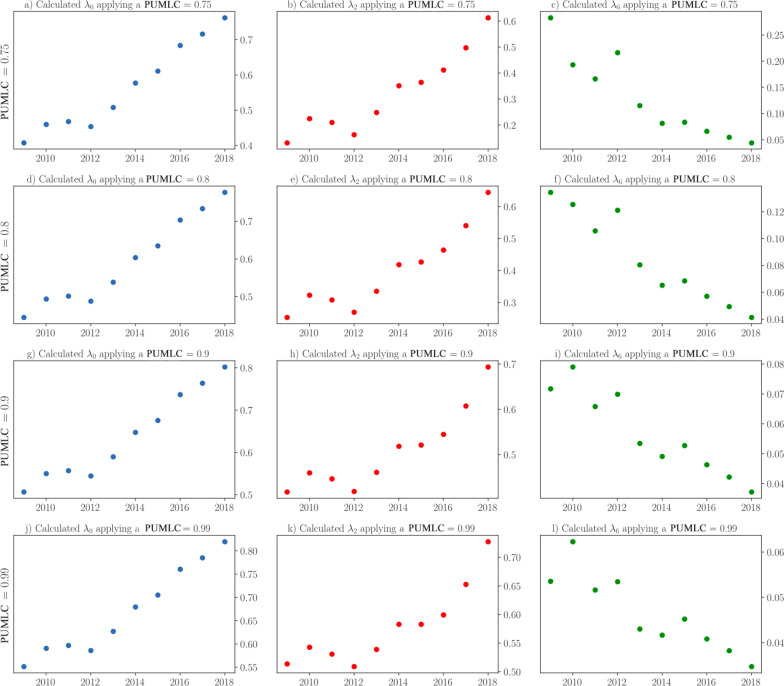
Parameterized probabilities of *λ*_0_, *λ*_2_ and *λ*_6_ in relation to a PUMLC = 80% over the years of data used to train the model. *λ*_0_ is the probability of a case of MS being diagnosed during PC, *λ*_2_ is the probability of a pregnant woman diagnosed during PC receiving adequate treatment and *λ*_6_ is the probability of a diagnosed and treated case of MS leading to CS

Figure 3 depicts, as an example, the variation in $\lambda_{0} , \lambda_{2} ,$ and $\lambda_{6}$ over the years of the data used to train the model for $PUMLC = \left\{ {0.75, 0.85, 0.95} \right\}$. Thus, we observed an upward trend for the probability that a mother reported to have syphilis may be diagnosed during PC ($\lambda_{0}$) for all investigated PUMLC values. From the group of women reported as diagnosed with syphilis during PC, the probability of receiving adequate treatment ($\lambda_{2}$) also revealed an increasing trend. However, the probability of a diagnosed and treated case of MS leading to CS ($\lambda_{6}$) showed a decreasing trend.

### Simulation evaluation

The SPN model developed in this work was expected to determine the number of MS cases and the firing rates in a given interval used as input data. Using output data, it returned the number of CS cases. As seen before, the set of firing rates can be represented in its entirety by only four probabilities, namely, $\lambda_{0} , \lambda_{2} ,$ and $\lambda_{6}$ and the PUMLC, where all others are written as functions of these probabilities.

In the previous subsection, we obtained a set of regressions that allowed for the prediction of $\lambda_{0} , \lambda_{2} ,$ and $\lambda_{6}$ for the test years with the PUMLC as a parameter. Equally important is that when we parameterized the PUMLC value, there were four different regression techniques for each of the three probabilities calculated here. Thus, we obtained a total of 64 arrangements for each tested $PUMLC = \left\{ {0.75, 0.8, 0.85, 0.9, 0.95} \right\}$. The analytical calculation, hence deterministic, was used for each of the 64 possible arrangements. The process of reproducing the results achieved in this paper was preserved.

To evaluate the output data, we used the mean absolute percentage error (MAPE) [27] as a performance metric to determine the best configuration of regression techniques to predict the number of CS cases across all years tested. To validate the stochastic characteristic of the SPN model, 100 independent experiments were performed with optimal initial settings. Thus, the *T*-test [28] was used to verify whether there was a statistically significant difference between the distribution of the SPN results and analytical calculation results.

**Fig. 4:**
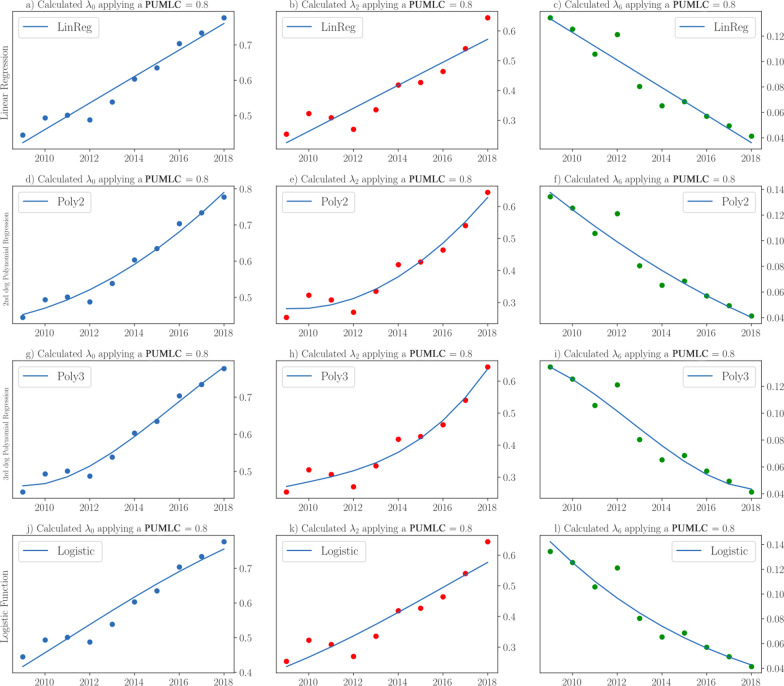
Four different regressions for the probabilities of *λ*_0_, *λ*_2_ and *λ*_6_ when applying a PUMLC = 80%

The *T*-test assumes that the sample data have a normal distribution, so we used the Shapiro–Wilk test [29] with a significance level of $0.05$ to verify the normality of the distributions of 100 independent experiments from the SPN simulations (Fig. 4).

Subsequently, we identified that all samples had *p*-values greater than $0.05$, showing normal data. Then, the two-tailed *T*-test was applied with a significance level $= 0.05$. The null hypothesis is rejected in this test if the *p*-value is less than $0.05$. Such a hypothesis is that the mean of the distribution $\mu_{0} = x$, where $x$, in this particular case, denotes the value obtained through analytical calculation. The alternative hypothesis is that $\mu_{0} \ne x$.

## Results

This section presents the results of (1) the evaluation of the regression arrangements, (2) the SPN simulations in different scenarios, and (3) the statistical tests used to compare the analytical and stochastic results developed in this paper.

Table 4 summarizes the best regression arrangements for estimating the probabilities for each PUMLC value observed. It is worth mentioning that the optimization metric chosen to determine the best arrangement was the mean absolute percentage error.

**Table 4:** Summary of the model evaluation using regression techniques

PUMLC	$\lambda_{0}$	$\lambda_{2}$	$\lambda_{6}$	2019 accuracy (%)	2020 accuracy (%)	MAPE (%)
0.75	Logistic	Linear	Linear	97.23	99.97	1.3987
0.8	Poly 3	Poly 3	Poly 3	97.69	99.95	1.1801
0.85	Poly 3	Poly 2	Logistic	95.99	93.15	5.4303
0.9	Poly 3	Poly 2	Poly 2	97.20	95.91	3.4481
0.95	Poly 3	Poly 2	Poly 2	98.24	98.25	1.7553

PUMLC, probability of an untreated MS case leading to CS case

The accuracy for each year is also presented, as the numbers we found were consistently above 93%. In this context, the lowest MAPE of 1.18%—namely, the most successful result—occurred for a PUMLC of 80%. In this regard, the accuracy values for 2019 and 2020 were 97.69% and 99.95%, respectively, while to estimate the firing rates $\lambda_{0} , \lambda_{2} ,$ and $\lambda_{6}$, the best regression was the 3rd-degree polynomial (see Table 4 for more details). In contrast, the highest MAPE of 5.43%, was observed for a PUMLC of 85%, with the best arrangement of regressions among the 64 possible arrangements as follows: linear, logistic, 2nd degree polynomial and 3rd degree polynomial to estimate the values of $\lambda_{0} , \lambda_{2} ,$ and $\lambda_{6}$, respectively.

Since the data described in Table 4 were obtained analytically, producing deterministic results, it was essential to compare them with the distributions obtained through the SPN simulations. Thus, Table 4 presents the results of the *t*-test used for such a purpose.

In general, all *p*-values presented in Table 5 had a significance level greater than $0.05$. Thus, we could not reject the null hypothesis and therefore concluded that there was no statistically significant difference between the predicted SPN values and the analytical results from calculating the number of CS cases. Consequently, Table 5 details the results that allowed us to deduce that SPNs are adequate for modeling the relationship between reported maternal and congenital syphilis cases from data available on SINAN.

**Table 5:** *T*-test results for the similarity between analytical congenital syphilis (CS) and the stochastic Petri net (SPN) predictions

PUMLC	2019		2020	
	CS analytic	*p*-value	CS analytic	*p*-value
0.75	25,137	0.4766	14,970	0.0635
0.80	25,192	0.5485	15,004	0.1991
0.85	24,838	0.8948	15,421	0.9613
0.90	24,986	0.7767	14,719	0.7955
0.95	25,051	0.3371	14,757	0.9256

PUMLC, probability of an untreated MS case leading to CS case

**Fig. 5:**
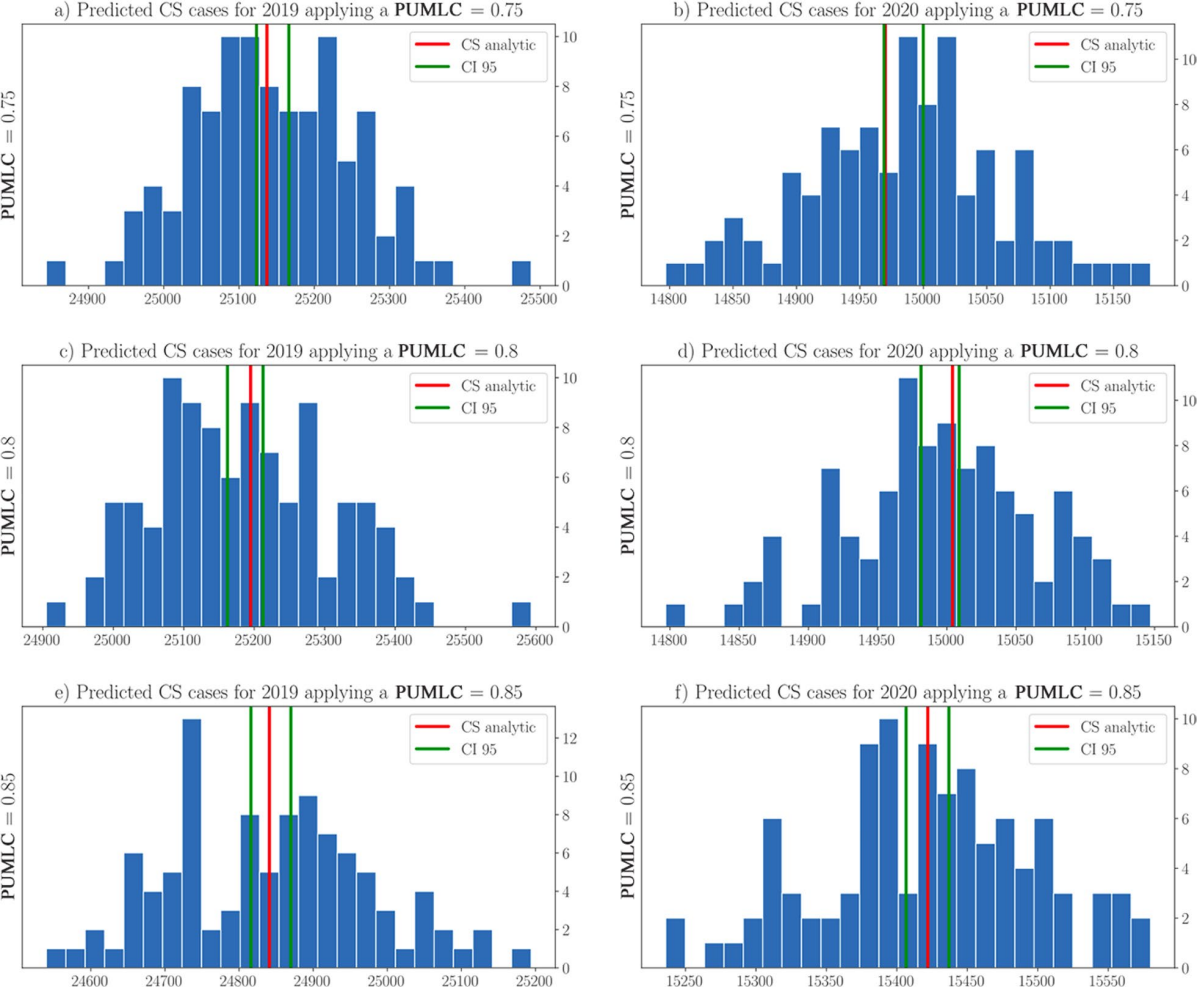
Distributions of predicted CS cases by the SPN when applying a *PUMLC* = {0.75, 0.80, 0.85}

**Fig. 6:**
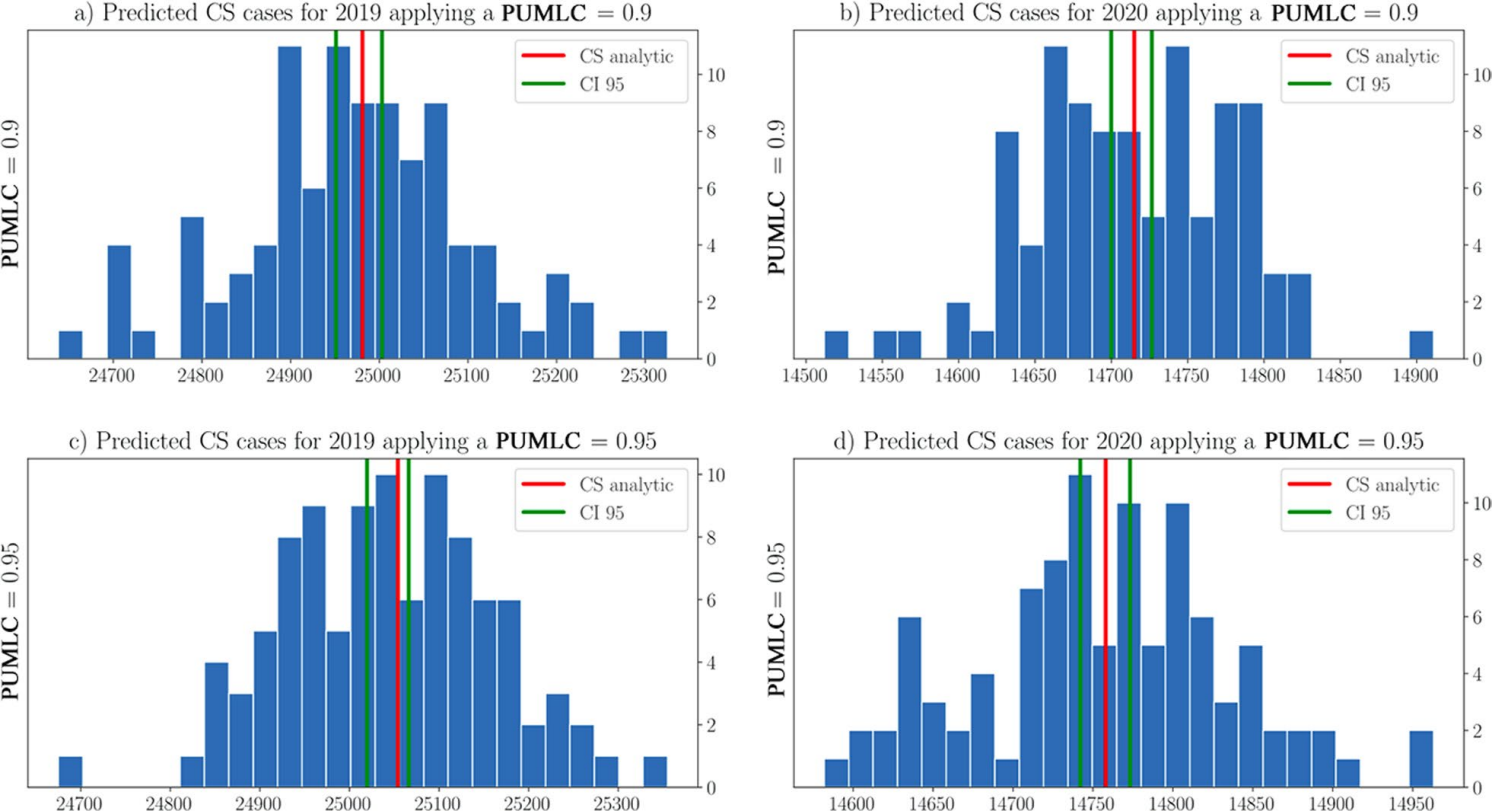
Distributions of predicted CS cases by the SPN when applying a *PUMLC* = {0.90, 0.95}

To illustrate the performance of the SPN graphically, Figs. 5 and 6 show the histograms of the distributions of the predicted CS cases obtained by the simulations. In addition, the 95% confidence intervals, shown in green lines, and the analytical value for CS, in red lines, were plotted. Figure 5a shows the histogram with 100 independent experiments using the parameter $PUMLC = 0.75$ for 2019. Figure 5d used $PUMLC = 0.8$ but targeted 2020. As one can see, the analytical values were within the confidence interval for all scenarios achieved. These results corroborated the findings of the statistical tests shown in Table 5 and therefore validated the analysis presented in Table 4.

## Discussion

In this study, it was possible to simulate the CS cases from the number of cases of MS with a percentage accuracy of at least 98.81%. Accordingly, this model achieved a favorable result when predicting real-world data using the diagnosis rates and adequate maternal treatment during PC as the parameters. The result of the test set of our model, using the Poly 3 regression functions, indicated an accuracy of 97.69% in 2019 and 99.95% in 2020 (see Table 4). CS is a global health burden that has drawn the attention of the World Health Organization (WHO). This United Nations agency presented a guide that included integrated processes and criteria for validation of the elimination of mother-to-child transmission (EMTCT) of HIV and syphilis [30].

Furthermore, several countries have adopted measures to eliminate MTCT, including Brazil [10]. The countries that succeeded in the EMTCT developed strategies to ensure universal and equitable antenatal care services, including HIV and syphilis screening tests and free-of-charge medical care for pregnant women [31, 32]. Brazil has followed similar strategies. Nevertheless, coordinating response efforts is an obstacle for the MoH since the country has continental dimensions, a large population, and enormous regional inequalities.

This has hampered efforts to reach the EMTCT. To succeed in the EMTCT, Brazilian policy-makers and stakeholders must develop strategies according to the characteristics of each region [33, 34].

In 2018, the Brazilian Federal Government launched a national strategy entitled the”Syphilis No!” Project (SNP) through the MoH. The SNP has been developing a bold campaign to raise the population’s awareness concerning syphilis testing and treatment. In addition, the project also aims to address the stigmas around STIs, especially syphilis [35, 36]. As part of the SNP, the MoH has delivered training processes to health care workers, from public health managers to frontline health practitioners. Furthermore, the project collaborates with national and international universities to strengthen research on syphilis and bolster technologies and information systems, such as the SINAN, which can help fight against this century-old ailment.

While it is clear that many processes exhibit deterministic behavior, it may be simpler to describe these phenomena using macroscopic probabilistic assumptions. This is usually done because the details are not entirely known, and even when they are, their inclusion can lead to unnecessarily complex models [37, 38]. In addition, a probabilistic approach can be advantageous since it provides sufficient precision by producing more general results and may aid in the study of sensitivity to parameter variations [39].

Taken together, this model may be helpful in improving strategic planning for effective response efforts in vulnerable populations and may contribute to the eradication of CS. According to the WHO agenda, the strategy to eradicate CS requires improving surveillance systems and identifying new indicators to detect and adequately treat infected pregnant women early in gestation [2, 40].

In this vein, the development and implementation of new tools to predict the occurrence of acquired and congenital syphilis and other STIs plays a vital role in epidemiological surveillance and may contribute to effective problem-oriented decision-making. Therefore, this paper proposed and explained a mathematical model based on SPNs to predict the relationship between reported maternal and congenital syphilis cases, considering maternal serological status and adequate treatment during PC based on accurate data obtained from governmental health professionals.

## Conclusion

This study introduced a stochastic Petri net to predict the notifications of CS cases based on the notifications of syphilis in pregnant women and the probability of maternal diagnosis and adequate treatment during PC. Therefore, this model can be used by public health managers to elaborate on and execute effective measures to control the transmission of acquired and congenital syphilis. Finally, our study may potentially support goals and actions related to three distinct aspects: the number of syphilis cases during pregnancy, the provision of screening tests, and the adequate treatment of all infected patients, mainly pregnant women, contributing to a reduction in CS cases.

## Data Availability

All data used in this manuscript are publicly available data from the Information Technology Department of the Brazilian National Health System (DATASUS) database at the following link: https://datasus.saude.gov.br/acesso-a-informacao/doencas-e-agravos-de-notificacao-de-2007-em-diante-sinan/.

## Abbreviations

CS: Congenital syphilis
EMTCT: Elimination of mother-to-child transmission
MAPE: Mean absolute percentage error
MoH: Ministry of Health
MS: Maternal syphilis
PC: Prenatal care
PUMLC: Probability of an untreated maternal syphilis case leading to congenital syphilis case
RCMCN: Ratio of congenital to MS case notifications
SINAN: Notifiable Diseases Information System
SPN: Stochastic Petri net
STD: Sexually transmitted disease
WHO: World Health Organization
