# Correction to: Stochastic Petri net model describing the relationship between reported maternal and congenital syphilis cases in Brazil

**DOI:** 10.1186/s12911-022-01812-x

**Published:** 2022-03-25

**Authors:** Ricardo A. M. Valentim, Gleyson J. P. Caldeira-Silva, Rodrigo D. da Silva, Gabriela A. Albuquerque, Ion G. M. de Andrade, Ana Isabela L. Sales-Moioli, Talita K. de B. Pinto, Angélica Espinosa Miranda, Leonardo J. Galvão-Lima, Agnaldo S. Cruz, Daniele M. S. Barros, Anna Giselle C. D. R. Rodrigues

**Affiliations:** 1 Laboratory of Technological Innovation in Health, Federal University of Rio Grande do Norte, Natal, Brazil; 2 State Secretariat of Public Health of Rio Grande do Norte, Natal, Brazil; 3 Postgraduate Program in Infectious Diseases, Federal University of Espírito Santo, Vitória, Brazil; 4 Digital Metrópole Institute, Federal University of Rio Grande do Norte, Natal, Brazil

## Correction to: BMC Med Inform Decis Mak (2022) 22:40. 10.1186/s12911-022-01773-1

Following publication of the original article [1], it was reported that reference 35 was mistakenly published as a duplicate of reference 13. The correct reference is given here [2] and the original article [1] has been updated.
